# Clustered Cystic Changes in Long-Term Follow-Up Thin-Section Computed Tomographic Findings in Fibrotic Nonspecific Interstitial Pneumonia

**DOI:** 10.1155/2024/6665568

**Published:** 2024-02-14

**Authors:** Masanori Akira, Narufumi Suganuma

**Affiliations:** ^1^Department of Radiology, National Hospital Organization (NHO) Kinki-Chuo Chest Medical Center, 1180 Nagasonecho, Kita Ward, Sakai, Osaka 591-8025, Japan; ^2^Department of Environ Medicine, Kochi Medical School, Okochokohasu, Nankoku, Kochi 783-0043, Japan

## Abstract

**Objectives:**

The purpose of this study was to retrospectively assess cystic changes in findings on follow-up CT scans of patients with fibrotic nonspecific interstitial pneumonia (NSIP).

**Methods:**

The initial and last high-resolution CT scans of 58 patients with pathologically proven fibrotic NSIP were evaluated retrospectively. The median follow-up periods were 48 months (range, 12–183 months). The pattern, extent, and distribution of abnormal CT findings were compared with findings in the same region on previous and subsequent CT scans with a focus on cystic lesions.

**Results:**

Cystic lesions in a cluster were shown in 16 patients (28%) with fibrotic NSIP on the last CT scans. Focal clustered cysts were found in 5 cases and diffuse clustered cysts were seen in 11 cases. Focal clustered cysts mimicked honeycombing seen in usual interstitial pneumonia (UIP). Diffuse cysts were uniform in size in 7 of the 11 cases. Traction bronchiectasis in a cluster was seen in 3 of the 7 cases. The clustered cystic changes on CT during the course of NSIP mainly consisted of traction bronchiectasis and bronchiolectasis.

**Conclusions:**

Long-standing NSIP did not form honeycombing. The clustered cysts in patients with fibrotic NSIP were mainly remodeling of bronchiectasis.

## 1. Introduction

Nonspecific interstitial pneumonia (NSIP) represents one histologic subtype of idiopathic interstitial pneumonia. The high-resolution computed tomography (HRCT) feature of NSIP includes ground-glass opacity, reticulation, traction bronchiectasis, subpleural sparing, and no or minimal honeycombing [1–5]. The key HRCT features that favor the diagnosis of NSIP over usual interstitial pneumonia (UIP) are ground-glass opacity with overall extent being greater than the extent of reticulation, homogeneous lung involvement, subpleural sparing, upper lung irregular lines, and minimal honeycombing [4, 5]. Proximal bronchiectasis, in contrast to distal bronchiectasis, is also a sign of NSIP. The American Thoracic Society/European Respiratory Society/Japanese Respiratory Society/Latin American Thoracic Association guidelines require honeycombing for a specific CT diagnosis of UIP [6, 7]. Honeycombing without traction bronchiectasis can be a specific sign of UIP pattern. Distinction between NSIP and UIP is important because they are different in prognosis and treatment.

The follow-up CT of patients with NSIP is variable. Some improve with treatment, whereas others develop findings consistent with progressive fibrosis [8, 9]. Silva et al. [4] described that at follow-up, CT patients with initial CT findings compatible with NSIP progressed to findings compatible with idiopathic pulmonary fibrosis (IPF). However, whether NSIP progresses to UIP or not is unclear. The purpose of our study was to retrospectively assess the changes in findings on long-term follow-up CT scans of patients with fibrotic NSIP with a focus on cystic lesions.

## 2. Materials and Methods

The initial and last thin-section CT scans of 58 patients with pathologically proven fibrotic NSIP were evaluated retrospectively. Patient selection was done by reviewing the medical records of all patients who had received a histologic diagnosis of NSIP on the basis of surgical lung biopsy between 2002 and 2018 in our hospital. They all were idiopathic. Patients with a defined connective tissue disease (CTD) or a known cause of interstitial lung disease (e.g., hypersensitivity pneumonitis and drug-induced lung disease) were excluded. Patients who revealed established CTD during the follow-up were excluded in this study. Among these patients, patients who underwent serial high-resolution CT (HRCT) examinations during a follow-up period of more than one year were included in this study. In all patients, the histologic diagnoses of fibrotic NSIP were made by consensus agreement between at least two pathologists, by using the combined American Thoracic Society and European Respiratory Society Consensus Classification criteria [6]. Organizing pneumonia (OP) and fibrotic OP were excluded.

Only those patients with a final clinical diagnosis of idiopathic fibrotic NSIP were included. The study sample consisted of 31 men and 27 women, with a mean age of 57 years (range, 37–80 yr). The interval between the initial and follow-up CT scans ranged from 12 – 183 months (median, 48 months) in the patients. Approval for this study was obtained from the Institutional Clinical Research Ethical Board. The Institutional Clinical Research Ethical Board waived the need of informed consent. All methods were carried out in accordance with relevant guidelines and regulations (Declaration of Helsinki).

The treatments were started after surgical lung biopsy. Eleven patients received prednisone alone, nine patients received prednisone and cyclosporine A, 10 patients received prednisone and azathioprine, 16 patients received prednisone and cyclophosphamide, and 12 patients received prednisone, azathioprine, and cyclophosphamide.

### 2.1. CT Assessment

The HRCT examinations were performed with 1-mm or 1.5-mm collimation at 10-mm intervals from the apex of the lung to the diaphragm. The scans were obtained with the patient in the supine position at full inspiration and were reconstructed using a high-spatial-frequency algorithm. The HRCT scans (initial and follow-up) were randomized and reviewed by two independent thoracic radiologists, and the findings were agreed upon by consensus. The CT findings were interpreted on the basis of the recommendations of the nomenclature committee of the Fleischner Society [10]. Honeycombing is defined as clustered cysts suggesting dilatation of peripheral airspace due to surrounded fibrosis. The observers evaluated the overall extent of parenchymal abnormalities and the extents of ground-glass opacity, consolidation, reticulation, and honeycombing that were present in both lungs to determine the percentage of lung parenchyma occupied by the disease. The lungs were divided into six zones and each zone was evaluated separately. The six areas of the lung included the upper zones, above the level of the carina; the middle zones, between the level of the carina and the level of the inferior pulmonary veins; and the lower zones, under the level of the inferior pulmonary veins. The score was based on the percentage of the lung parenchyma that showed evidence of the abnormality and was estimated to the nearest 10% of parenchymal involvement. The overall percentage of lung involvement was calculated by averaging the six lung zones. The extent of traction bronchiectasis was evaluated by counting the number of segments that showed evidence of traction bronchiectasis. Ten segments in the right lung and 9 segments in the left lung were evaluated. The traction bronchiectasis score was recorded as 0 to maximum 19 [11]. In addition, the pattern, extent, and distribution of abnormal CT findings were compared with findings in the same region on previous and subsequent CT scans.

All statistical analyses were performed using SPSS statistical software (version 12.0J, 2003; SPSS, Inc., Chicago, IL). Group comparisons were made by using a paired *t*-test, or *χ*^2^ statistic. A *p* value of less than 0.05 was considered to indicate statistical significance.

## 3. Results

Clinical and demographic features at the initial CT scan are shown in Table 1. The parenchymal abnormalities on the initial and last CT scans of patients with NSIP are presented in Table 2. Overall extents of parenchymal abnormalities were 16.4 ± 10.5% on the initial CT and 20.4 ± 23.1% on the last CT in fibrotic NSIP (*p*=0.288).

**Figure 1:**
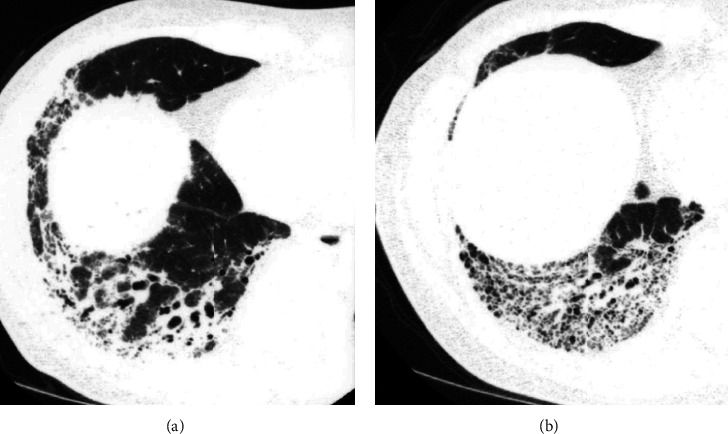
Sequential HRCT scans in a 62-year-old man with fibrotic NSIP. (a) Initial CT scan shows irregular consolidation with traction bronchiectasis and bronchiolectasis (arrows). (b) Follow-up scan obtained 54 months later shows more traction bronchiolectasis and fine cystic lesions. Pathologic examinations showed not organizing pneumonia but airspaces-filling mucinous exudates. Consolidation was thought to be formed by prominent thickening of alveolar septa by collagen, volume loss of airspaces, and airspaces-filling mucinous exudate.

**Figure 2:**
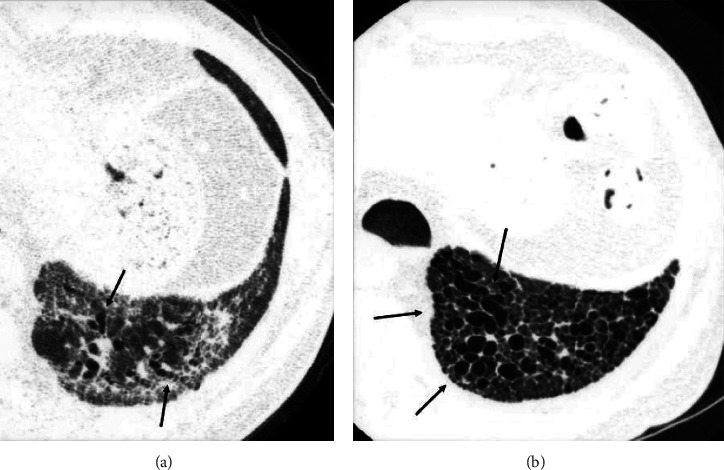
Sequential HRCT scans in a 58-year-old man with fibrotic NSIP. (a) Initial CT scan shows ground-glass opacity with reticulation and traction bronchiectasis (arrows). (b) Follow-up scan obtained 36 months later shows that traction bronchiectasis has progressed and formed clusters (arrows).

**Figure 3:**
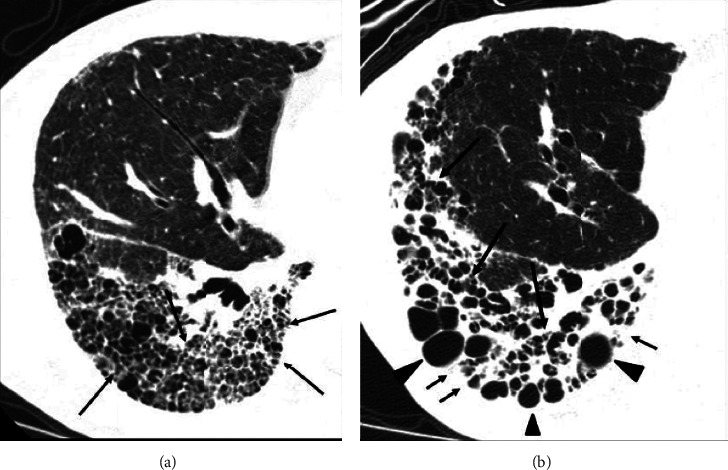
Sequential HRCT scans in a 74-year-old woman with fibrotic NSIP. (a) Initial CT scan shows traction bronchiectasis and bronchiolectasis (arrows) and cysts. The subpleural cysts seldom protuberate toward the extrapulmonary region. (b) Follow-up scan obtained 25 months later shows that many small cysts are collapsed and dense increased opacities have appeared. Traction bronchiectasis (long arrows) has progressed and some cysts have become larger (arrowheads). There are dense increased opacities in the subpleural region (short arrows).

**Figure 4:**
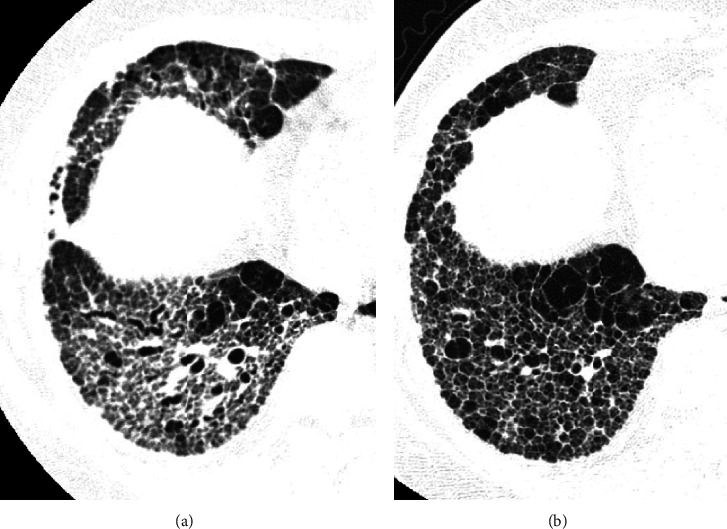
Sequential HRCT scans in a 52-year-old woman with fibrotic NSIP. (a) The initial CT scan shows reticular opacities with traction bronchiectasis. (b) Follow-up scan obtained 40 months later shows coarse reticular opacities with low attenuation areas and traction bronchiectasis. Most of the polygonal areas are not typical cystic airspaces. Pulmonary vessels can be seen in some polygonal areas. Air space enlargement may be occurring regularly.

The last CT showed clustered cysts in 16 (28%) patients in fibrotic NSIP. In these 16 cases, the median follow-up periods were 42 months (range, 26–112 months). Focal clustered cysts were found in 5 cases and diffuse clustered cysts were seen in 11 cases. Focal clustered cysts mimicked honeycombing seen in UIP which is subpleural. They were found in the right middle portion of the lung in 1 case, in the left middle portion of the lung in 2 cases, and in the unilateral basal lung in 2 cases. Diffuse cysts were uniform in size in 7 cases and traction bronchiectasis in a cluster was seen in 3 of the 7 cases. Diffuse cystic lesions in a cluster in fibrotic NSIP were classified into four patterns: fine cystic lesions suggesting traction bronchiolectasis (*n* = 4) (Figure 1), traction bronchiectasis in a cluster (*n* = 3) (Figure 2), traction bronchiectasis and bronchiolectasis and large cysts (*n* = 2) (Figure 3), and traction bronchiectasis and reticulation with low attenuation (*n* = 2) (Figure 4). They consisted of traction bronchiectasis and bronchiolectasis. Very few small cysts in NSIP protuberate toward the extrapulmonary region, whereas cysts in UIP often protuberate toward the side of the extrapulmonary region.

## 4. Discussion

In our study, cystic lesions in a cluster were shown in 28% with fibrotic NSIP on the last CT scans. Silva et al. [4] suggested that there were two separate NSIP subgroups, with one subgroup representing NSIP progressing to a pattern that resembles IPF and one subgroup that maintains the overall features of NSIP even with progression of fibrosis. She described that 28% of patients with initial CT findings suggest NSIP progressed to findings suggestive of IPF at follow-up CT [4]. Lasti et al. [12] reported that a minority of NSIP patients exhibited early deterioration despite treatment, and this was predictive of an IPF-like course to a fatal outcome. Another study shows that in NSIP, patients compatible with UIP on the initial thin-section CT diagnosis had a worse prognosis [8]. Some authors [13, 14] described that in patients with IPF, the pattern of abnormality characteristic of NSIP was found on initial CT.

**Figure 5:**
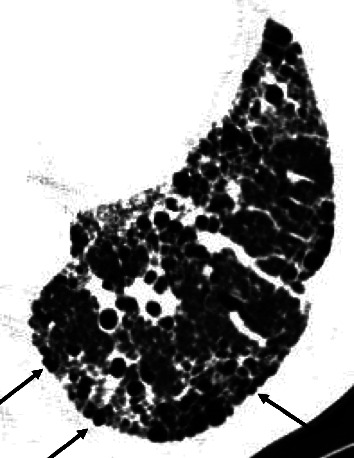
HRCT of honeycombing in UIP. Honeycomb cysts in the peripheral portion of the lung protuberate toward the side of extrapulmonary region (arrows).

Honeycombing is defined as clustered cysts suggesting dilatation of peripheral airspace due to surrounded fibrosis [15]. Honeycombing consists not only of dilatation of peripheral airspaces but also of dilatation of bronchioles [16]. In advanced cases with fibrotic NSIP, numerous bronchiolectasis can occur and they may be difficult to be distinguished from honeycombing suggesting dilatation of peripheral airspace due to surrounded fibrosis. Moreover, in advanced cases with fibrotic NSIP, there are reticular opacities more than ground-glass opacities in extent, so the criteria of predominant reticular opacity more than ground-glass opacity are not used to discriminate UIP from long-standing fibrotic NSIP. Honeycomb formation is slow and less extensive in fibrotic NSIP than in IPF [8]. Some cysts in fibrotic NSIP were not honeycomb cysts. Traction bronchiectasis in a cluster sometimes is a mimicker of honeycombing. In NSIP, clustered cysts are mainly traction bronchiolectasis and bronchiectasis. Traction bronchiolectasis with a connection of a bronchus has a centrilobular location, whereas traction bronchiolectasis, of which honeycombing consists in UIP, is seldom connected with a bronchus, as dilated bronchioles are elongated and distorted [15]. Traction bronchiolectasis in NSIP are uniform in size and appear homogeneous. This change may reflect the temporal homogeneity of NSIP noted pathologically [17]. Also, traction bronchiectasis in NSIP is less distorted than that in UIP and can be seen over long distances longitudinally. In my experience, even small honeycomb cysts in the peripheral portion of the lung sometimes protuberate toward the side of the extrapulmonary region (Figure 5), whereas the subpleural cysts in NSIP seldom protuberate toward the extrapulmonary region.

Our study has several limitations. Our HRCT scans were not performed in volumetric CT acquisitions. Volumetric CT acquisitions made more precise assessment of our findings. Our classification is descriptive and other patterns may be found. However, the purpose of our study is not classification. Focal clustered cysts may not be honeycombing but bronchiectasis which is subpleural. We enrolled patients with NSIP diagnosis, having at least 2 examinations with more than 1 year of interval, so that the evaluation could be heterogeneous.

## 5. Conclusions

Less than 30% cases with fibrotic NSIP showed clustered cysts as the previous reports. The clustered cystic changes on CT during the course of NSIP mainly consisted of traction bronchiectasis and bronchiolectasis. In the reviewed sample, long-standing NSIP does not form honeycombing. The clustered cysts in patients with fibrotic NSIP are mainly remodeling of bronchiectasis.

## Figures and Tables

**Table 1:** Baseline clinical and demographic features of patients.

Age, yr	57 ± 2.3
Male/female	31/27
Smoking, current/ex/never	10/20/28
Duration of symptoms, mo, median (range)	2.3 (0–48)
Pulmonary function	
FVC, % predicted	74.5 ± 16.9
D_Lco_, % predicted	58.5 ± 18.4
PaO_2_, mmHg	72.4 ± 11.3

D_Lco_ = diffusion capacity of carbon monoxide; ex = ex-smoker.

**Table 2:** Computed tomography findings in initial and last scans in patients with fibrotic NSIP (*n* = 58).

CT findings	Initial CT scan	Last CT scan	*p* value
Overall extent^*∗*^	16.4 ± 10.5	20.4 ± 23.1	0.288
GGO extent^*∗*^	11.9 ± 8.5	12.7 ± 17.7	0.777
Reticulation extent^*∗*^	7.0 ± 6.9	10.2 ± 10.7	0.090
Traction bronchiectasis score^✝^	4.4 ± 3.9	7.2 ± 5.1	0.001
Consolidation^*∗*^	2.3 ± 3.5	0.4 ± 1.2	0.001
Clustered cysts or honeycombing^⁑^	3 (5)	6 (28)	0.018
Clustered cysts or honeycombing extent^*∗*^	0.1 ± 0.5	2.0 ± 5.3	0.024

^*∗*^Data are mean percentage of lung parenchyma ± standard deviation. ^✝^Data are mean score ± standard deviation. ^⁑^Data are numbers of patients, with percentages in parentheses.

## Data Availability

No underlying data were collected or produced in this study.

## Abbreviations

NSIP: Nonspecific interstitial pneumonia
CT: Computed tomography
HRCT: High-resolution computed tomography
UIP: Usual interstitial pneumonia
IPF: Idiopathic pulmonary fibrosis.
